# Reducing the costs per patient by increasing the volume of cataract surgery

**Published:** 2022-12-16

**Authors:** Jonathan Malcolm, Ahmed Bako

**Affiliations:** 1 International Centre for Eye Health, London School of Hygiene and Tropical Medicine, London, UK.; 2 Department of Ophthalmology, Specialist Hospital, Sokoto, Nigeria.

**When eye units increase their cataract output, a small increase in the outlay (for consumables and IOLs) can drastically increase income and/or reduce costs for patients.**

**Figure:**
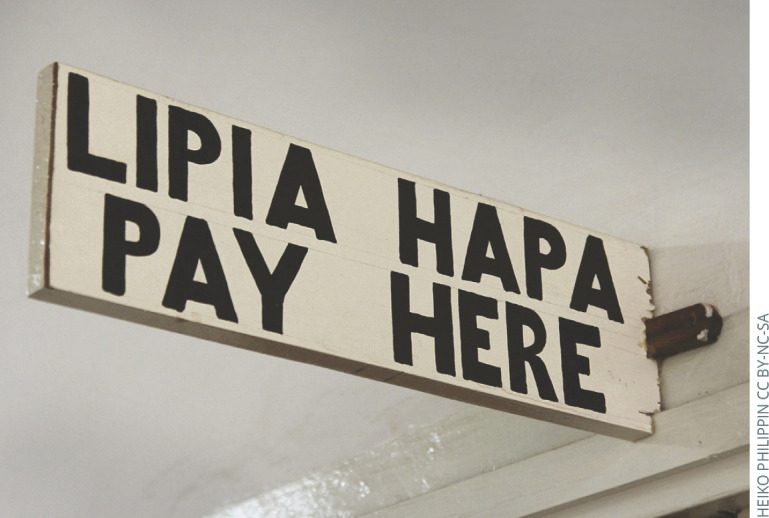
The cost of cataract surgery can be a major barrier. **TANZANIA**

Cataract is the leading cause of avoidable blindness worldwide.^1^ Since the burden of cataract blindness is greatest in the communities who are least able to afford eye care, cost is a major barrier to patients accessing cataract surgery.^2–4^ The financial barriers to patients accessing cataract surgery may be greater in rural areas, as additional travel, accommodation, and food costs are often incurred.^5^ Lack of access to cataract surgery can be financially devastating, often resulting in reduced economic potential because of vision impairment.^6^ Thus, designing more accessible and affordable cataract services is essential for tackling inequalities and overcoming poverty.

The aim of this article is to discuss high-volume cataract surgery as a strategy for lowering the cost of cataract surgery per patient. High-volume cataract surgery does not have an absolute definition, but is often considered as a service that carries out significantly more cataract operations than centres in the surrounding area.^7^

Cataract surgery costs can be divided into the costs of **consumables** (such as intraocular lenses, medication, anaesthetics, and disposables) and the costs of **infrastructure and salaries** (Figure 1).^7^ Each cataract operation uses approximately the same amount of consumables, therefore the yearly cost of consumables varies in line with the number of cataract operations performed in that year.

**Figure 1:**
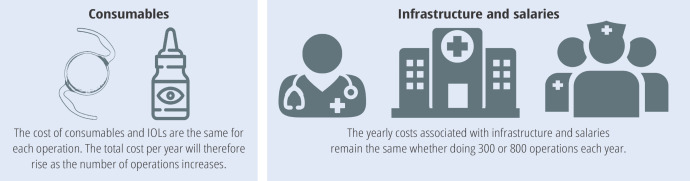
The costs associated with cataract surgery

The cost of **infrastructure and salaries** is typically larger than the costs of **consumables** and must be paid regardless of the number of cataract operations performed each year. Examples of **infrastructure and salary** costs include staff salaries, equipment, cleaning, and building maintenance.

Although increasing the yearly number of cataract operations (the cataract volume, or output) will increase the total yearly cost of consumables, the cost of infrastructure and salaries remains fixed. By carrying out more operations per year, the infrastructure and salary costs – which can make up the bulk of the total cost of surgery in smaller centres – is therefore shared between more patients, **bringing down the cost per patient** for an individual cataract operation. Increasing the cataract volume also enables further reductions in the cost per operation through taking advantage of ‘economies of scale’ such as bulk purchasing of consumables: by buying a large number of items at once, lower prices could be negotiated, further reducing the cost per operation.

## Growing your surgical output

A key assumption of high-volume cataract surgery is that most cataract services have unused capacity. Estimates of East African cataract services suggest that, although surgeons currently perform fewer than 300 operations each per year, they could perform 500 to 800 per year if improvements were made to management systems.^8^ This would have to be matched by increasing the number of patients who come for surgery, as detailed elsewhere in this issue and the previous issue on community engagement. Since staff salaries are a major fixed cost, optimising the number of operations performed per surgical day by theatre teams is an effective strategy for reducing the cost per eye.^8,9^

## Hypothetical example: a cataract service with an annual output of either 500 or 800 operations

**Figure 2:**
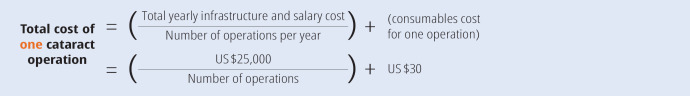
Calculating the cost of one cataract operation

Here is a hypothetical example of a cataract service where the yearly infrastructure and salary cost is $25,000 per year, and the cost of consumables for one cataract operation is approximately $30. The **total cost** of **one** cataract operation can be calculated by dividing the total yearly cost of infrastructure and salaries ($25,000) by the number of operations per year, then adding the consumables cost (see the formula in Figure 2).

**Figure 3:**
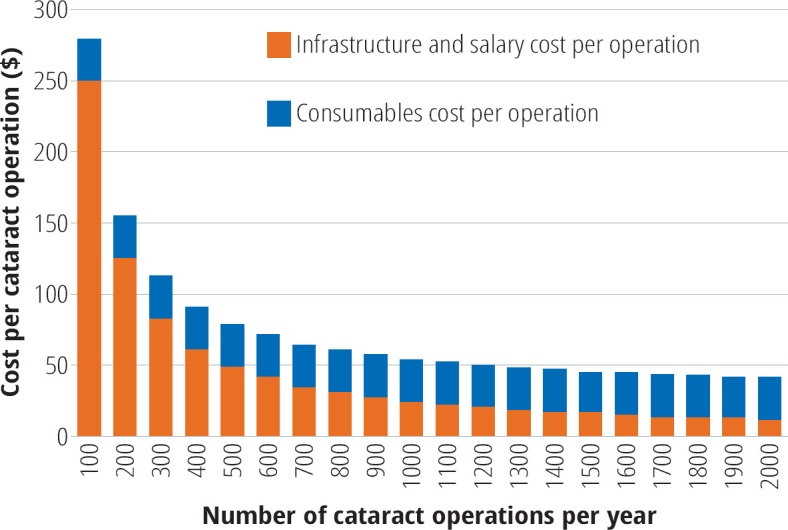
Changes in the cost of a single cataract operation as the number of operations per year increases

Figure 3 is also based on our hypothetical example, and shows how the cost per operation reduces as the number of cataract operations per year increases. Table 1 shows how the costs per operation is calculated for 500 and 800 operations, respectively.

**Table 1:** Cost per cataract operation for an annual cataract volume (operations per year) of 500 and 800, respectively

Annual cataract volume	500 operations per year	800 operations per year
Consumables cost per operation	$30	$30
Infrastructure and salary costs per operation	$25,000 ÷ 500 = $50	$25,000 ÷ 800 = $31.25
**Total outlay per operation**	**$80**	**$61.25**

Taking the examples in Table 1, for 500 and and 800 operations per year, we can work out the costs (or outlay) and profit per operation.

Say the hospital charges patients $85 per cataract operation:

- If the annual cataract volume is 500 cataract operations per year, the outlay is $80 per operation ($30 for consumables + $50 for infrastructure and salaries). If the hospital charges $85 for cataract surgery, it makes a profit of $5 from each operation.
- If the annual cataract volume is 800 cataract operations per year, the outlay is $61.25 per operation ($30 for consumables + $31.25 for infrastructure and salaries). If the hospital charges $85 for cataract surgery, it makes a profit of $23.75 from each operation.

Table 2 shows the outlay and profit on an annual basis for a surgical volume of 500 and 800 operations per year. For 500 operations per year, the annual profit is $2,500, and for 800 operations per year, the profit is $19,000 per year.

Increasing the cataract output by 300 operations per year requires an additional outlay of $9,000 to cover the cost of the IOLs and consumables. But this is more than made up for by the increase in income from £2,500 to £19,000: an increase of £16,500.

**Table 2:** Annual cost, income and profit of a cataract service performing either 500 or 800 operations per year

Annual cataract volume	500 operations per year	800 operations per year
Consumables cost (annual)	$15,000	$24,000
Infrastructure and salary costs (annual)	$25,000	$25,000
**Total outlay**	**$40,000**	**$49,000**
Total income @ $85 per operation	$85 × 500 = $42,500	$85 × 800 = $68,000
**Total profit**	**$2,500**	**$19,000**

Additional profits generated by increasing the number of cataract operations could be used to subsidise patients who would otherwise struggle to afford surgery, or could be reinvested in services to make them more sustainable.
